# Deep learning for efficient reconstruction of highly accelerated 3D FLAIR MRI in neurological deficits

**DOI:** 10.1007/s10334-024-01200-8

**Published:** 2024-08-30

**Authors:** Luka C. Liebrand, Dimitrios Karkalousos, Émilie Poirion, Bart J. Emmer, Stefan D. Roosendaal, Henk A. Marquering, Charles B. L. M. Majoie, Julien Savatovsky, Matthan W. A. Caan

**Affiliations:** 1 Department of Biomedical Engineering & Physics, Amsterdam UMC Location University of Amsterdam, Meibergdreef 9, 1105 AZ Amsterdam, The Netherlands; 2 Amsterdam Neuroscience, Brain Imaging, Amsterdam, The Netherlands; 3 Department of Radiology and Nuclear Medicine, Amsterdam UMC Location University of Amsterdam, Meibergdreef 9, Amsterdam, The Netherlands; 4 Fondation Rothschild Hospital, 29 Rue Manin, Paris, France

**Keywords:** MRI, Accelerated imaging, Deep learning, Neurological deficits, Image resolution

## Abstract

**Objective:**

To compare compressed sensing (CS) and the Cascades of Independently Recurrent Inference Machines (CIRIM) with respect to image quality and reconstruction times when 12-fold accelerated scans of patients with neurological deficits are reconstructed.

**Materials and Methods:**

Twelve-fold accelerated 3D T2-FLAIR images were obtained from a cohort of 62 patients with neurological deficits on 3 T MRI. Images were reconstructed offline via CS and the CIRIM. Image quality was assessed in a blinded and randomized manner by two experienced interventional neuroradiologists and one experienced pediatric neuroradiologist on imaging artifacts, perceived spatial resolution (sharpness), anatomic conspicuity, diagnostic confidence, and contrast. The methods were also compared in terms of self-referenced quality metrics, image resolution, patient groups and reconstruction time. In ten scans, the contrast ratio (CR) was determined between lesions and white matter. The effect of acceleration factor was assessed in a publicly available fully sampled dataset, since ground truth data are not available in prospectively accelerated clinical scans. Specifically, 451 FLAIR scans, including scans with white matter lesions, were adopted from the FastMRI database to evaluate structural similarity (SSIM) and the CR of lesions and white matter on ranging acceleration factors from four-fold up to 12-fold.

**Results:**

Interventional neuroradiologists significantly preferred the CIRIM for imaging artifacts, anatomic conspicuity, and contrast. One rater significantly preferred the CIRIM in terms of sharpness and diagnostic confidence. The pediatric neuroradiologist preferred CS for imaging artifacts and sharpness. Compared to CS, the CIRIM reconstructions significantly improved in terms of imaging artifacts and anatomic conspicuity (p < 0.01) for higher resolution scans while yielding a 28% higher SNR (p = 0.001) and a 5.8% lower CR (p = 0.04). There were no differences between patient groups. Additionally, CIRIM was five times faster than CS was. An increasing acceleration factor did not lead to changes in CR (p = 0.92), but led to lower SSIM (p = 0.002).

**Discussion:**

Patients with neurological deficits can undergo MRI at a range of moderate to high acceleration. DL reconstruction outperforms CS in terms of image resolution, efficient denoising with a modest reduction in contrast and reduced reconstruction times.

**Supplementary Information:**

The online version contains supplementary material available at 10.1007/s10334-024-01200-8.

## Introduction

Time is of utmost importance in diagnosing neurological deficits such as multiple sclerosis (MS) [1, 2] and tumors [3] and initiating prompt treatment in patients with confirmed acute ischemic stroke (AIS). MS and tumor patients are commonly recalled to the clinic for repeated magnetic resonance imaging (MRI) to monitor disease progression. In the case of AIS, MRI plays a vital role in distinguishing between ischemic or hemorrhagic strokes or mimics [4, 5], visualizing any occlusions, and estimating the onset time [6, 7] and infarct core size [8]. The longer acquisition time required for an MRI examination than for computed tomography (CT) raises concerns about delaying immediate management and treatment decisions during emergencies in the case of a stroke. Given these considerations, exploring ways to accelerate MR sequences currently utilized in diagnosing neurological deficits is necessary.

In recent decades, there have been important advancements in accelerating clinical MRI. Initially, iterative reconstruction techniques such as SENSE [9] and GRAPPA [10] were proposed for parallel imaging. Compared with parallel imaging, compressed sensing (CS) [11] involves accelerating MRI through iterative reconstruction of irregularly undersampled data to a much greater degree. When imaging neurological deficits, most sequences can benefit from CS. One study used a combination of CS and SENSE to accelerate 3D T1-echo-spoiled gradient echo and T2-FLAIR sequences up to five times [3]. Others have also used CS and SENSE to accelerate the time-of-flight MR angiography (TOF-MRA) sequence by approximately ten times [12, 13].

Iterative image reconstruction techniques have two main limitations that hinder their use in a fast-paced clinical environment. First, iterative reconstruction may yield a prolonged reconstruction time. Especially for high-resolution images obtained after zero-filling, the reconstruction times can exceed the measurement times, which limit the clinical workflow. Second, the quality of the CS-reconstructed image may deteriorate when the acceleration factor increases [1]. Thus, novel reconstruction techniques applied to neurological deficits should ideally decrease scanning and reconstruction times while preserving image quality.

Deep learning (DL) can accelerate the imaging time by using graphical processing units (GPUs) for reconstruction while allowing for efficient denoising of the data. For example, one study used DL to reduce the scanning time by approximately 60% when reconstructing data [14]. Additionally, DL has exhibited promising results in reconstructing MRI data with pathologies [2, 15, 16], making it a valuable tool for clinical applications.

Physics-informed DL methods learn how to solve the inverse problem of accelerated MRI reconstruction from the data [17–19]. The objective is to map the undersampled k-space measurements to a denoised image. This approach benefits from generalizing well to modalities not seen during the network training. Recently, the Cascades of Independently Recurrent Inference Machines (CIRIM) were proposed, which balances efficiency and network complexity, and is fast with excellent denoising and generalization capabilities [20]. This could make this network a good candidate for use in neurological deficits. The CIRIM was shown to outperform CS reconstruction in terms of commonly computed metrics, i.e., structural similarity and the peak signal-to-noise ratio. In fast and potentially time-critical imaging settings, CS may thus render inferior image quality, increasing the need for improved image reconstruction under these conditions. Furthermore, an extensive clinical evaluation of this method is still lacking.

This work aims to achieve highly accelerated imaging and fast reconstruction in diagnosing patients with neurological deficits. We evaluate the image reconstruction performance of the previously proposed CIRIM in a representative clinical dataset. This dataset consists of highly accelerated (12X) 3D T2-FLAIR images obtained as part of routine clinical practice and includes data from fifty-seven patients with neurological deficits, stroke, tumors, and multiple sclerosis (MS). Compressed sensing (CS) is the reference reconstruction method used in the clinic to which we compare our CIRIM method. Challenges lie in the dataset’s inhomogeneity and preservation of pathologies unseen during training. Since the dataset was acquired as part of the clinical routine, no fully sampled scans are available; these scans take too long to acquire clinically. Therefore, the CIRIM is trained on a different dataset and compared to CS in terms of reconstruction times and image quality, which are scored both subjectively and objectively. Subdisciplines in radiology may have different requirements in terms of image quality and scanning time. We compare the perceived image quality rated by radiologists with diverse specializations, i.e., intervention neuroradiology and pediatric neuroradiology.

## Materials and methods

### Patients and ethics

The data for this retrospective study were routinely collected in our hospital (anonymized for review). All patients included in this study (n = 62, 34 females) came to the hospital as part of the clinical routine, including patients with stroke (n = 8), other vascular pathologies (n = 6), multiple sclerosis (relapsing–remitting MS, n = 10; progressive MS, n = 3; undefined MS, n = 4), tumors (n = 8), and Meniere's disease (n = 3). The mean age was 53 ± 14 (range: 9 to 88) years. The sample size was chosen such that subjects over a broad age range with a spectrum of diseases were included. All the data were anonymized prior to analysis. Informed consent was not required according to the IRB.

### Data acquisition

Patient data were consecutively acquired on a 3 T Philips Ingenia Elition scanner equipped with a 32-channel head coil between 08/2021 and 02/2023. The scan parameters of the T2-FLAIR sequence varied and were in the following ranges: field of view (FOV) from 249 × 249x180 to 251 × 251x180 mm, scanning matrix from 216 × 174x120 to 240 × 251x180, zero-filled reconstruction matrix from 336 × 336x240 to 528 × 528x360, acquisition resolution from 1.05 × 1.00x1.00 to 1.15 × 1.43x1.50 mm^3^, and reconstruction resolution from 0.48 × 0.48x0.50 to 0.74 × 0.74x0.75 mm^3^. The other parameters were TR = 8000 ms, TE = 311 ms, TI = 2400 ms, turbo factor = 186, and scan time = 1m52s to 3m00s. As per standard of care, the data were prospectively undersampled with a variable density mask with a radial shutter to a factor of 12. Sensitivity-reference scan data were obtained for coil sensitivity estimation. Raw data were retained in archive per clinical routine and exported in addition to on-scanner reconstructions in DICOM format.

### Data (pre)processing

The raw data were preprocessed in a custom pipeline in MATLAB (version R2019b, MathWorks). Preprocessing for parallel-imaging CS (PICS) and CIRIM reconstruction was identical. The FLAIR k-space data were loaded, phase and offset-corrected, and sorted with MRecon (version 4.4.4, GyroTools). Oversampling was removed in the readout direction, and the matrix was zero-filled to match the original output resolution, leading to an eightfold increase in matrix size. The sensitivity-reference scan was upsampled and brought into alignment with the FLAIR scan. Sensitivity maps were calculated with caldir (range 50) implemented in the BART toolbox [21]. Five subjects were discarded due to excessive motion artifacts, in which there was no exclusion bias toward a particular diagnostic label, resulting in a dataset of fifty-seven (n = 57) subjects.

### Parallel-imaging compressed sensing (PICS)

Offline CS reconstructions were performed via the BART toolbox. We used the PICS algorithm with a ℓ1-wavelet sparsity transform. The regularization factor was heuristically set to 0.5 to balance artifacts and noise, for a maximum of 60 iterations.

### Cascades of independently recurrent inference machines (CIRIM)

**Fig. 1:**
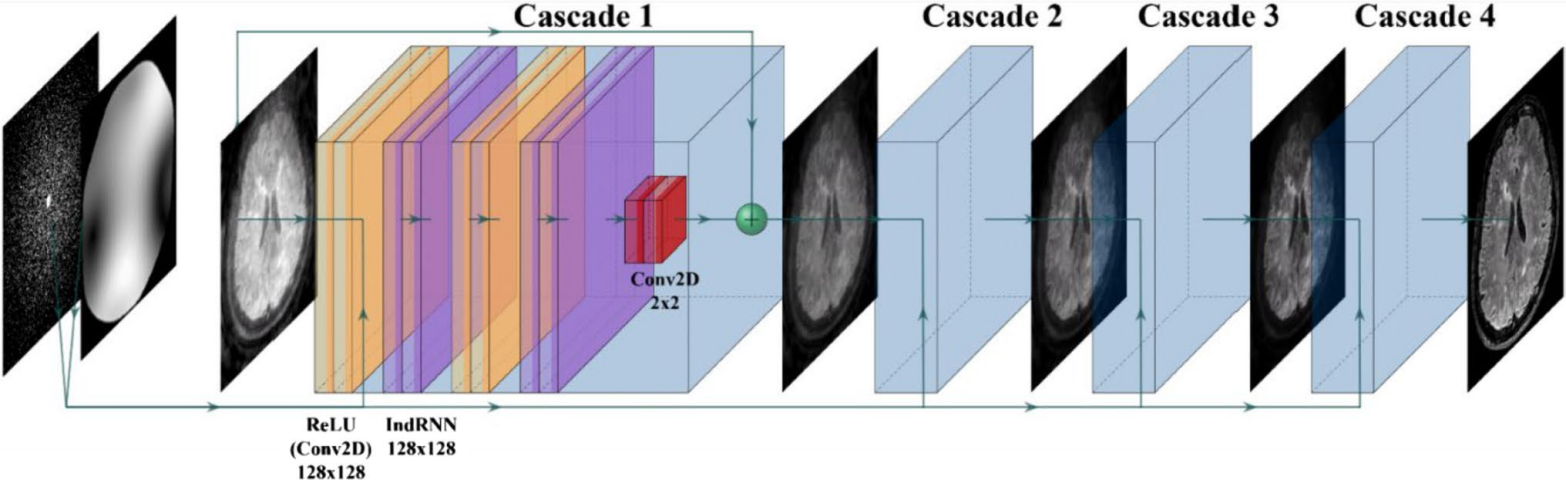
Schematic showing the architecture of the Cascades of Independently Recurrent Inference Machines (CIRIM) with four cascades. From left to right: raw k-space data and accompanying sensitivity maps are used to create an initial estimate entered into an IRIM block for calculating the gradient to update the image. An IRIM block consists of subsequent convolutional layers activated by a rectified linear unit (ReLU), recurrent layers (IndRNN), and a final convolutional layer. Four identical IRIM blocks are connected into cascades that share features but no parameters

For DL reconstruction, we trained a CIRIM on fully sampled 3D T1-weighted data of healthy volunteers, retrospectively undersampled twelve times from a 2D variable density Poisson distribution. Previous work has shown that a network trained on T1-weighted data can generalize well to unseen FLAIR images [20]. Training data were acquired on a 3.0 T Philips Ingenia scanner (Philips Healthcare, Best, The Netherlands), and comprised magnetization-prepared rapid gradient echo (MPRAGE) scans, no acceleration, an isotropic resolution of 1.0 mm^3^ and a FOV of 256 × 240 mm^2^. The training set consisted of ten subjects (approximately 2000 slices), and the validation set consisted of one subject (approximately 200 slices). No cross-validation was performed during training. Rather, this study serves as an independent study with an external validation test dataset. An overview of the network architecture is shown in Fig. 1. The hyperparameters of the network were selected as follows. The number of channels was set to 128 for the recurrent and convolutional layers, the number of time steps was set to 8, and the number of cascades was set to 4. Additionally, we adopted and implemented a new stable backend using PyTorch Lighting 1.6.0 with floating-point 16 precision for fast reconstruction times. Model parameters were initialized randomly. The code is available online at https://github.com/wdika/atommic.

### Reconstruction time

The reconstruction time was measured as the total time taken for reconstructing a 32-channel volume of size 432 × 432x278. Notably, when performing a reconstruction with the BART toolbox, there is a small overhead per slice of writing a temporary file and deleting it. For a fair comparison, we accumulated this overhead time, approximately three seconds, and subtracted it from the final reconstruction times. The measurements were repeated three times to ensure precision.

Reconstructions were performed offline on an Nvidia Tesla V100 GPU card with 32 GB of memory.

### Expert ratings

All the reconstructions were stored in DICOM format for subjective rating of image quality. Two experienced interventional neuroradiologists (J.S., B.E.) with 23 and 17 years of experience and one experienced pediatric neuroradiologist (S.R.) with 14 years of experience were asked to subjectively rate the CIRIM and CS images on multiple categories. The raters were blinded to the diagnosis of the cases whose images were processed for the study. The raters were only asked to review the FLAIR sequence. They scored images side-by-side on multiple categories on a 1 to 5 image quality scale. The scores were as follows: 1 for non-diagnostic quality, 2 for poor quality, 3 for acceptable quality, 4 for good quality, and 5 for excellent quality. The order of the reconstruction methods was randomized, and the raters remained unaware of the method used and the patients’ clinical information. Inspired by previous work, five scoring categories were adopted. Imaging artifacts related to aliasing resulting from image acceleration, ranging from excessive artifacts that severely degrade images to no artifacts present. Perceived spatial resolution referred to image sharpness and the ability to discern small structures down to the voxel level sharpness, ranging from unacceptable, extreme blur levels to a high level of detail at the native level of the defined spatial resolution. Anatomic conspicuity ranged from being unable to discern (small) anatomical and pathological structures to perfect identification of structures. Diagnostic confidence summarized the certainty in the diagnosis of pathology, e.g., a lesion, on a scan, ranging from being unable and highly uncertain in diagnosis to perfect ability to diagnose a scan. Image contrast referred to the relative difference in the intensity of known tissue types and pathology ranging from no contrast visible to extremely good contrast [3, 22]. After the individual rating of the data, a review meeting was held with the readers, in which selected subjects with discrepancies in reading scores were re-evaluated.

### Quantitative analyses

Since a fully sampled scan of the patient data is lacking, we calculated self-referenced quantitative measures of image quality using MRI Quality Control (MRIQC) [23]. Specifically, we selected the following set of metrics that we deemed relevant for the task of image reconstruction: coefficient of joint variation (CJV) [24], signal-to-noise ratio, a quality index (QI_1_) of the proportion of voxels corrupted by artifacts [25], the entropy focus criterion (EFC), being the Shannon entropy of voxel intensities as an indication of ghosting and blurring induced by head motion [26], the foreground to background energy ratio (FBER), being the mean energy of image values within the head relative to outside the head [27], and the full width at half maximum (FWHM) of the spatial distribution of the image intensity values in units of voxels [28]. To assess the dependency of FWHM on SNR, Gaussian noise was added post hoc to one randomly selected CIRIM reconstruction and FWHM was recalculated.

To study the effect of acceleration factors on the reconstruction of patient data containing pathology, we trained a CIRIM on FLAIR scans adopted scans from the FastMRI dataset [29]. The training set consisted of 344 scans and the validation set consisted of 107 scans. The model was trained on a range of acceleration factors from 4 × to 10x. Details on the dataset and training are reported elsewhere [20]. For evaluation, we computed the structural similarity (SSIM) index over the entire validation set for all acceleration factors. Furthermore, we selected a subset of 10 patients for which labeled lesion locations were available [30]. For all acceleration factors, we computed the contrast ratio (CR) as (I_lesion_-I_WM_)/I_WM_ with I_lesion_ and I_WM_ being the median intensities in bounding boxes in the lesion and in white matter, respectively. We computed repeated-measures ANOVA to test for an effect of the acceleration factor on the SSIM and CR values.

In 10 randomly selected patients with a visible lesion of heterogeneous origin, a region of interest was manually annotated within the lesion and in white matter proximal to the lesion. CR was computed in both the CIRIM and the PICS reconstructions. A Wilcoxon signed-rank difference test was performed.

### Statistics

Statistical analyses were performed using SciPy [31]. The statistical significance threshold was set at p < 0.01 for all tests. Bonferroni correction for multiple comparisons was used when necessary. A one-sample Wilcoxon signed-rank test was used to determine whether the expert scores significantly preferred one over the other reconstruction method. A paired t-test was used to determine whether the SNR differed significantly between methods. Probabilistic ordinal linear regression was performed to evaluate the interaction effect of higher image resolution on improved rating scores, depending on the reconstruction method used. The voxel volume was used as image resolution metric. For each reconstruction method and per patient group, post hoc one-sample Wilcoxon signed-rank tests were used to determine significance at a Bonferroni-corrected threshold over multiple scoring categories.

## Results

**Fig. 2:**
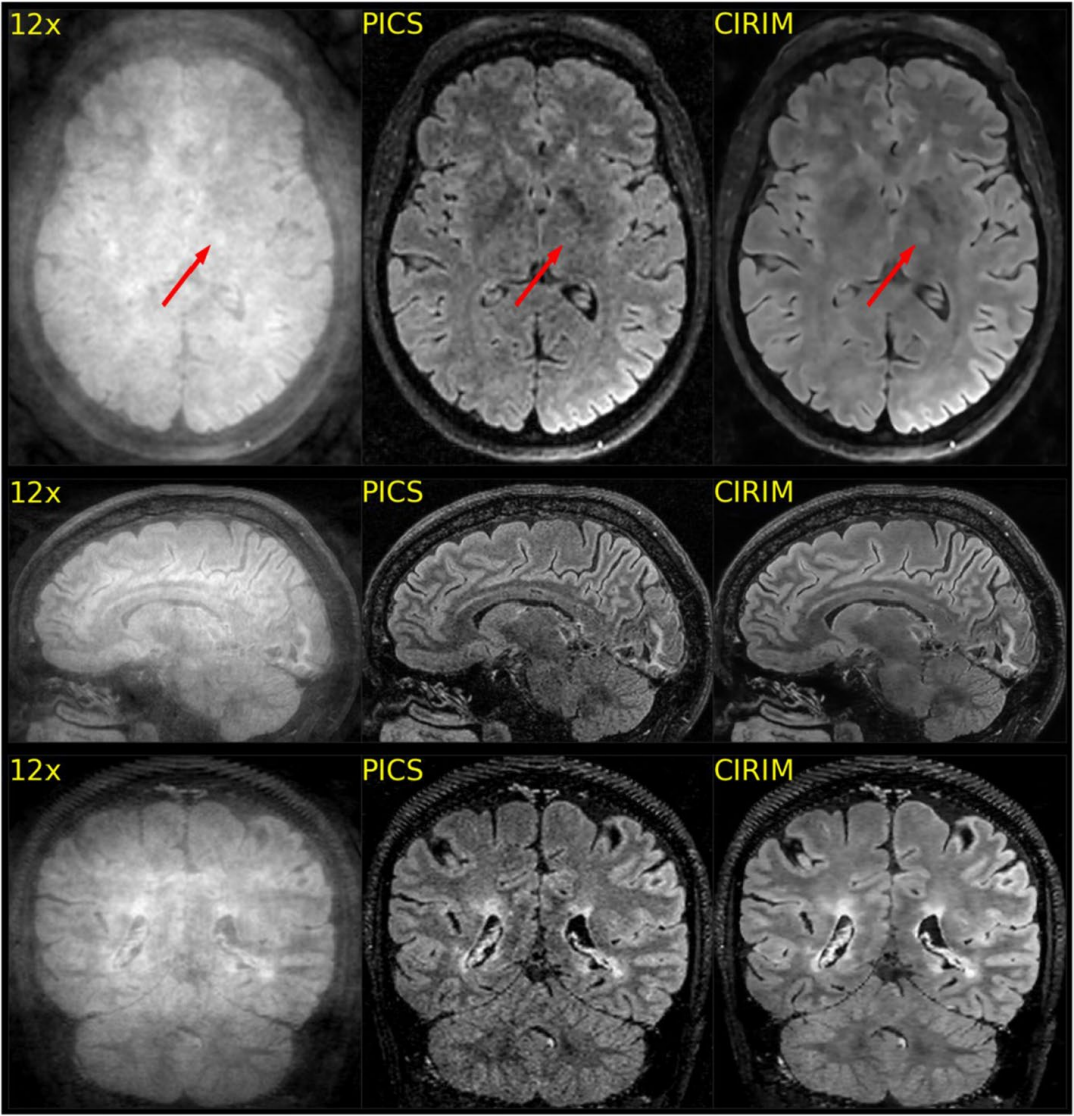
Reconstructions of 12 times accelerated FLAIR scans for three different subjects, where the CIRIM was able to generate better image quality. In the top row, PICS could not accurately depict the left internal capsule lesion (example indicated by the arrow), whereas the CIRIM preserved the contrast

**Fig. 3:**
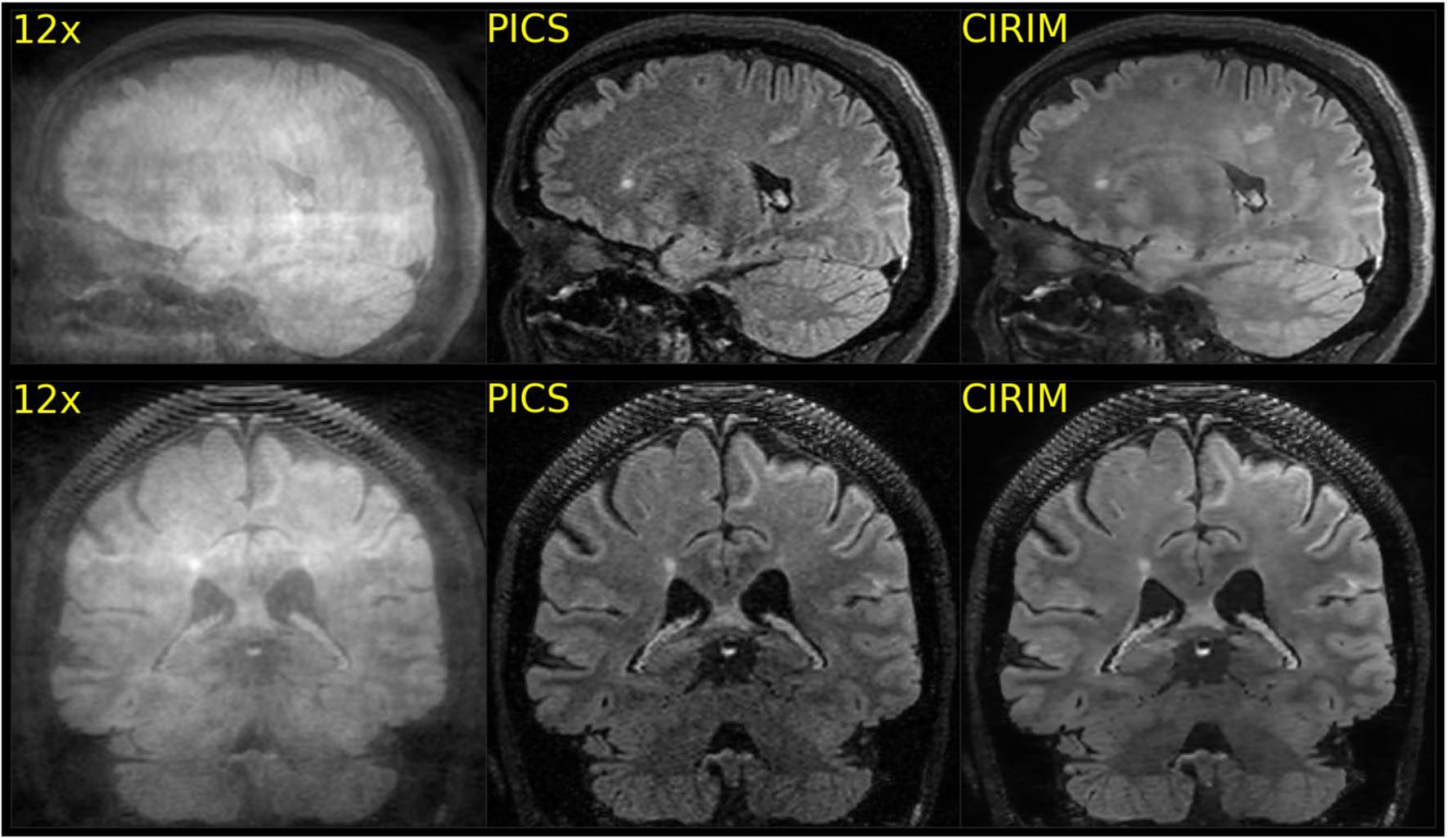
Reconstructions of 12 times accelerated FLAIR scans of two subjects, where the CIRIM and PICS provided high-quality reconstructions but were interpreted differently by two raters in a side-by-side comparison. Rater 2 interpreted the CIRIM reconstructions as having sharper edges, whereas Rater 3 interpreted the grainier PICS reconstructions as resulting in increased sharpness

**Fig. 4:**
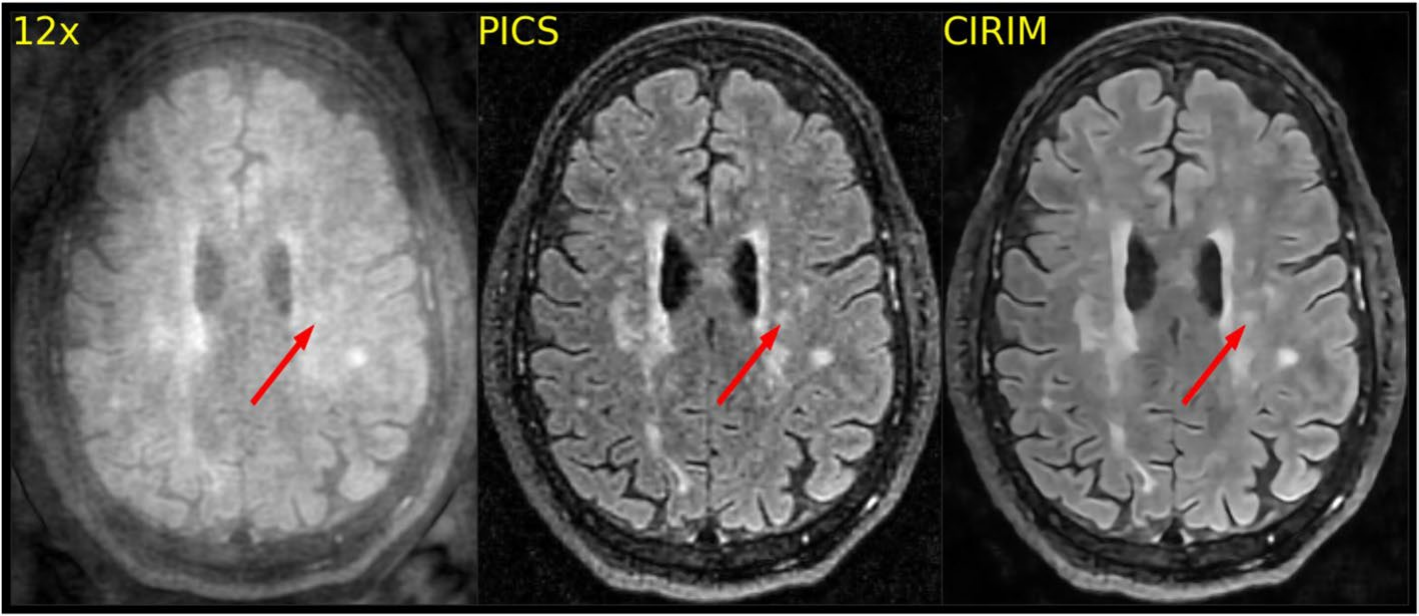
Reconstructions of a 12 times accelerated FLAIR scan, where the CIRIM yielded a more blurred reconstruction than PICS did for small T_2_ high signal intensities among patients with small vessel disease (example indicated by the arrow)

In total, the data of 57 subjects were complete and could be successfully reconstructed via the PICS and the CIRIM network. Figure 2 shows selected example images in which efficient denoising of the CIRIM compared with PICS can be seen. The CIRIM was also able to accurately reconstruct structures where PICS failed, such as the left internal capsule in the first example (Fig. 2-top row). Figure 3 depicts example images where the raters did not clearly prefer one method. Ratings varied in terms of imaging artifacts and sharpness. In rare cases, PICS was preferred over the CIRIM in terms of image sharpness, as illustrated in Fig. 4.

The raters significantly preferred the CIRIM over PICS in most cases, as shown in Table 1. For imaging artifacts, Rater 2 and Rater 3 significantly preferred the CIRIM (mean ± SD = 3.8 ± 0.6 vs. 3.4 ± 0.5, p < 0.01, and 2.9 ± 0.4 vs. 2.2 ± 0.6, p < 0.01, respectively), whereas Rater 1 preferred PICS (4.6 ± 0.7 vs. 4.1 ± 0.8, p < 0.01). With respect to sharpness, Rater 2 preferred the CIRIM (4.1 ± 0.6 vs. 3.4 ± 0.5, p < 0.01), Rater 3 preferred PICS (2.8 ± 0.5 vs. 2.3 ± 0.5, p < 0.01), and Rater 1 showed no significant difference. Rater 1 and Rater 3 preferred the CIRIM for anatomic conspicuity (4.5 ± 0.7 vs. 3.3 ± 0.8, p < 0.01, and 3.0 ± 0.5 vs. 2.6 ± 0.6, p < 0.01, respectively) and contrast (4.8 ± 0.5 vs. 3.2 ± 0.6, p < 0.01, and 3.0 ± 0.5 vs. 2.5 ± 0.6, p < 0.01, respectively). While Rater 1 significantly preferred the CIRIM in terms of diagnostic confidence (4.7 ± 0.5 vs. 3.5 ± 0.8, p < 0.01), Rater 3 had no increased diagnostic confidence in the CIRIM after Bonferroni correction (2.9 ± 0.6 vs. 2.6 ± 0.7, p = 0.029).

**Table 1:** Subjective ratings of the clinical cohort from two expert interventional neuroradiologists, Raters 1 and 2, and one expert pediatric neuroradiologist, Rater 3, of 57 side-by-side CIRIM and PICS reconstructions

Category	Rater 1			Rater 2			Rater 3		
	Method (Mean ± SD)		p value	Method (Mean ± SD)		p value	Method (Mean ± SD)		p value
	PICS	CIRIM		PICS	CIRIM		PICS	CIRIM	
Imaging artifacts	4.6 ± 0.7	4.1 ± 0.8	< 0.001*	3.4 ± 0.5	3.8 ± 0.6	0.005*	2.2 ± 0.6	2.9 ± 0.4	< 0.001*
Perceived spatial resolution (Sharpness)	4.5 ± 0.7	4.5 ± 0.7	0.639	3.4 ± 0.5	4.1 ± 0.6	< 0.001*	2.8 ± 0.5	2.3 ± 0.5	< 0.001*
Anatomic conspicuity	3.3 ± 0.8	4.5 ± 0.7	< 0.001*	3.8 ± 0.6	3.9 ± 0.6	0.463	2.6 ± 0.6	3.0 ± 0.5	0.002*
Diagnostic confidence	3.5 ± 0.8	4.7 ± 0.5	< 0.001*	3.8 ± 0.6	4.1 ± 0.6	0.050	2.6 ± 0.7	2.9 ± 0.6	0.029**
Contrast	3.2 ± 0.6	4.8 ± 0.5	< 0.001*	3.9 ± 0.7	3.7 ± 0.7	0.088	2.5 ± 0.6	3.0 ± 0.5	0.003*

Images were scored from 1 to 5. A score of 1 indicates nondiagnostic quality, 2 poor quality, 3 acceptable quality, 4 good quality, and 5 excellent quality. A one-sample Wilcoxon signed-rank test was used to determine significance at a Bonferroni-corrected threshold of p = 0.05/5 = 0.01, indicated in bold

*SD* standard deviation

^*^Significant difference

^**^Not significant after Bonferroni correction

Rater 1 reported two illustrative multiple sclerosis (MS) cases where lesions were better visible in CIRIM reconstructions. In another patient, Rater 1 noted that the internal capsule was not visible on the PICS reconstruction. In contrast, the CIRIM resulted in better image quality (Fig. 2). Rater 3 reported that the CIRIM reconstruction was more blurred in 20 cases (scoring 2 on Sharpness): out of these cases PICS outscored the CIRIM by one point 14 times, and both scored equally on Sharpness six times. In one case, only the PICS reconstruction perceived by Rater 3 was more blurred (scoring 2 on sharpness). Rater 3 also stated that subtle MS lesions sometimes appeared slightly blurred, making it harder to discriminate them from artifacts. Raters 2 and 3 agreed that the CIRIM reconstructions were smoother than PICS reconstructions without apparent loss of detail. The interpretations of these two raters can be seen in Fig. 3. The figure shows high-quality reconstructions of both PICS and the CIRIM, where PICS produces grainier images, whereas the CIRIM results in smoother images. In a few selected cases, the grainy results of PICS resulted in higher sharpness scores than those of the CIRIM (Fig. 4). Patient group analyses revealed no significant effect of disease on rating scores. Higher image resolution yielded significantly better imaging artifacts (p < 0.01) and anatomic conspicuity (p < 0.01) rating scores in CIRIM reconstructions (Table 2). For PICS, no improvements were observed. The interaction effect between reconstruction method and resolution was non-significant (p > 0.05).

**Table 2:** Probabilistic ordinal linear regression for evaluating the effect of image resolution depending on the reconstruction method used

Category	Resolution			
	PICS		CIRIM	
	β [CI]	p	β [CI]	p
Imaging artifacts	0.098 [ − 0.287, 0.482]	0.619	0.528 [0.135, 0.922]	< 0.008*
Perceived spatial resolution (Sharpness)	0.222 [ − 0.166, 0.610]	0.262	0.503 [0.109, 0.896]	0.012**
Anatomic conspicuity	− 0.008 [ − 0.391, 0.375]	0.967	0.562 [0.163, 0.961]	< 0.006*
Diagnostic confidence	0.159 [ − 0.224, 0.541]	0.417	0.494 [0.098, 0.891]	0.015**
Contrast	0.262 [ − 0.122, 0.645]	0.181	0.483 [0.082, 0.884]	0.018**

A one-sample Wilcoxon signed-rank test was used to determine significance at a Bonferroni-corrected threshold of p = 0.05/5 = 0.01, indicated in bold

^*^Significant difference

^**^Not significant after Bonferroni correction

**Fig. 5:**
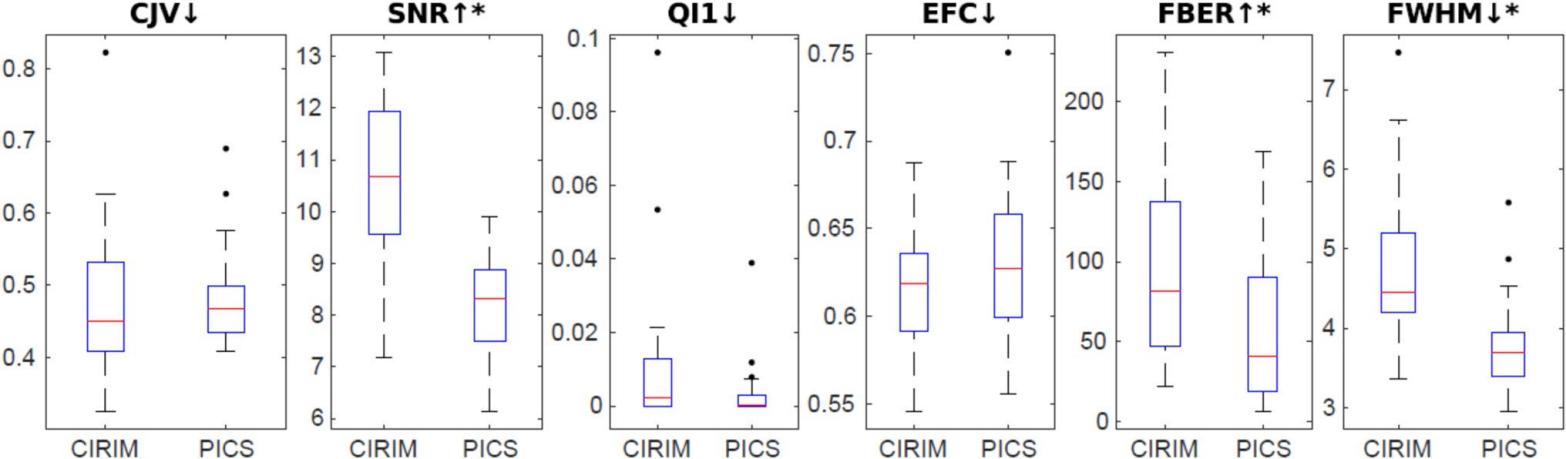
Quantitative self-referenced MRI Quality Control (MRIQC) metrics: coefficient of joint variation (CJV), signal-to-noise ratio (SNR), quality index 1 (QI1), entropy focus criterion (EFC), foreground to background ratio (FBER), full width at half maximum (FWHM). Arrows indicate better values. Significance is indicated with an asterisk (*) at *p* < 0.05 (c orrected)

Quantitative self-referenced MRI Quality Control (MRIQC) metrics are reported in Fig. 5.A. CIRIM reconstructions had a significantly higher signal-to-noise ratio (SNR) and foreground to background energy ratio (FBER), yielding an improved outcome. The full width at half maximum (FWHM) was significantly higher in CIRIM reconstructions, reflecting a worse scoring. Adding Gaussian noise to a CIRIM reconstruction, resulting in a lowering in SNR from 11 to 8.5, yielded a lower estimated FWHM of 3.8 instead of 4.4.

In terms of reconstruction times, CIRIM reconstructions were, on average, approximately five times faster (146 ± 1.7 s) than PICS reconstructions were (707 ± 20 s).

**Fig. 6:**
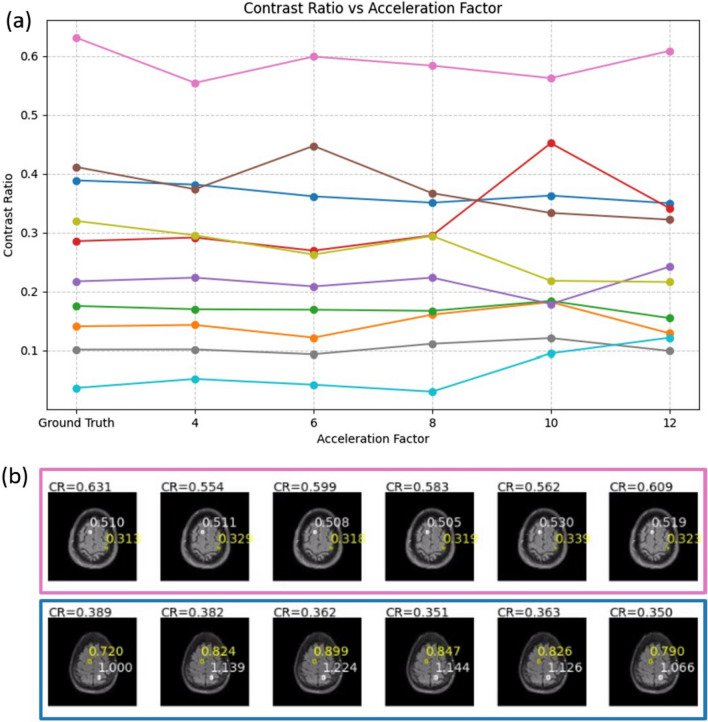
**a** Contrast ratio as a function of the acceleration factor for 10 FLAIR slices with an annotated white matter lesion in the FastMRI dataset. Different colors represent data from different subjects. **b** Selected reconstructions with bounding box colors matching the plotted lines in (**a**), and white and yellow bounding boxes positioned around the lesion and a selected white matter region

**Fig. 7:**
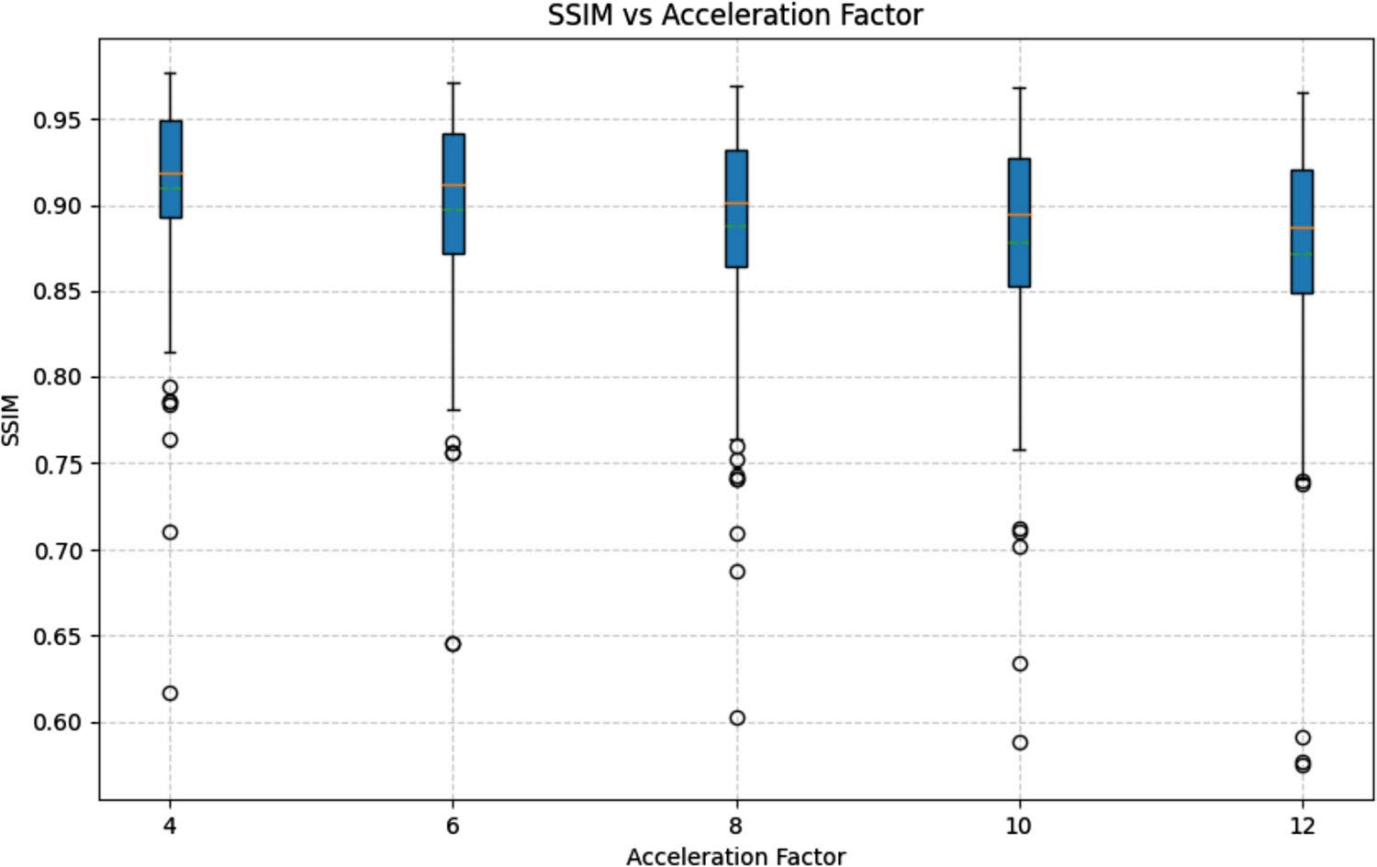
Structural similarity (SSIM) as a function of acceleration factor for the FastMRI validation set of 107 scans

Figure 6 shows contrast resolution (CR) as a function of the acceleration factor and highlights two example slices. No significant effect of the acceleration factor on the CR was observed (p = 0.92). The SSIM decreased by 4% from 0.92 down to 0.88 when increasing the acceleration factor from four-fold to 12-fold, which was a significant decline (p = 0.002), for which a plot is depicted in Fig. 7. Supplementary Fig. 1 displays example reconstructions and difference maps relative to the ground truth, illustrating that most inaccuracies are observed at tissue boundaries, and in effective denoising of artifacts present in the ground truth data.

**Fig. 8:**
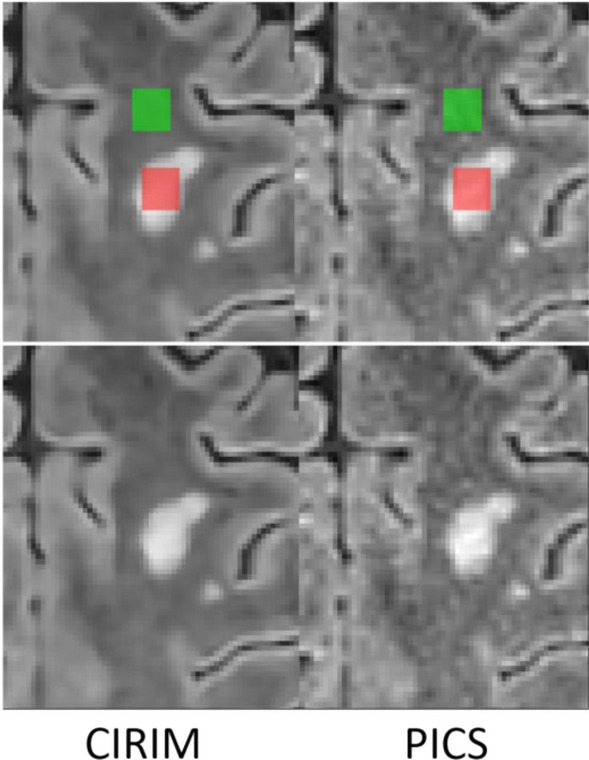
Manual annotation of a lesion (red) and proximal white matter (green) in CIRIM (left) and PICS (right) reconstructions

When comparing lesion contrast in manually annotated lesions in our data, on average, the CR is 5.8% lower in CIRIM reconstructions than in PICS reconstructions, decreasing from 1.17 to 1.10. This difference was significant (p = 0.04). Figure 8 illustrates that the CR is marginally lower but to a large extent preserved in CIRIM compared with PICS.

## Discussion

We demonstrated the value of reconstructing highly accelerated clinical FLAIR data with DL. The CIRIM could generalize well to heterogeneous clinical data that had not been previously reported, as it was trained on another distribution with another contrast (i.e., 3D-T1 scans of healthy volunteers). Rather than being explicitly trained on reconstructing a specific contrast or tissue, the physics-informed network has learned to efficiently denoise FLAIR images. Notably, obtaining fully sampled FLAIR data in patients is infeasible as it leads to excessive scanning times of up to 30 min at the current isotropic resolution, with associated imaging artifacts. Despite not being optimized for this type of data, the CIRIM retained its efficient denoising capacity in a dataset with a high degree of clinically desirable zero-filling.

Compared with the default PICS method, the CIRIM network’s denoising ability led to an almost 30% increase in SNR in brain tissue. In line with the improved calculated SNR, subjective metrics were primarily scored in favor of CIRIM. The increased SNR in CIRIM reconstructions led to significantly higher rating scores for imaging artifacts and anatomic conspicuity for higher-resolution images. While showing a smooth appearance, small features were still discernible in most images, which is clinically relevant [32]. The higher FWHM seen in CIRIM-reconstructions can be attributed to noise effects dominating the histogram distribution based on which this metric is computed. Image quality is thus maintained when reconstructing clinical data in a modality unseen during training, allowing for a reduction in scanning time or an increase in resolution while maintaining clinically acceptable image quality.

When embedding DL-enabled reconstruction methods in the clinic, it is interesting to note that the neuroradiologists, Raters 1 and 2, agreed on higher image quality in the CIRIM reconstructions than in the PICS reconstructions. On a more detailed level, a lower interrater agreement regarding sharpness and contrast in specific cases was observed. Specifically, Rater 1 significantly preferred CIRIM in terms of sharpness, suggesting that further denoised reconstructions yielded sharper edges than PICS did. Rater 2 perceived the denoising as a loss of spatial resolution or smoothness, resulting in a significant preference for PICS over CIRIM. Regarding image contrast, Rater 2 preferred CIRIM because of improved dealiasing of adjacent regions, leading to enhanced visibility of lesions. Notably, not all the raters were accustomed to reading data with a high acceleration factor (12 times) in their daily routine. Rater 3 reported that in the field of pediatric neuroradiology, image quality at a slightly less aggressive acceleration is to preferable. Previous work also reported mixed interrater agreement [3]. It is evident that CIRIM reconstructions differ significantly from iterative reconstruction algorithms, highlighting the need for good interaction and communication when implementing DL reconstruction methods in the clinic.

Another essential advantage of the model used is its ability to increase reconstruction speed, which is highly relevant in neurological deficits where acquisition speed, reconstruction times, and image quality need to be balanced. At large FOVs, PICS might be too slow for clinical use despite being deployed on fast GPUs. Image quality and reconstruction time can be traded within the CIRIM by choosing a different number of cascades than those used here. Compared with previous work, we increased the number of channels of the network from 64 to 128, aiming to improve image quality further.

The main protocol used in this study was designed to have a high acceleration factor of 12x. Other related works chose a more conservative acceleration factor in the range of 2 × to 4x [33]. This sequence was set up as multislice, allowing for acceleration along one phase-encoding dimension only. In contrast, here, a 3D FLAIR sequence is adopted with two phase encoding dimensions, allowing for much higher speed-up factors. Notably, this sequence is the standard of care in our clinic with compressed sensing reconstruction.

We intentionally designed the experiments to compare the performance of CIRIM and PICS on reconstruction alone and to do so offline (i.e., off-scanner). We wanted to exclude any on-scanner postprocessing to visually enhance the images, as this approach may be in place for some vendors. Postprocessing is typically performed with proprietary software. Thus, we disregarded this step to avoid an unbalanced comparison between methods. Moreover, leaving out post-processing makes the presented results more easily comparable with results from other vendors. However, in future research, it would be valuable to investigate the denoising capabilities of both CIRIM and on-scanner postprocessing filters for comparison purposes.

A few limitations need to be noted regarding the present study. In certain CIRIM reconstructions, a small amount of blur was introduced, possibly caused by the high acceleration factor (12-fold) and the resulting low intrinsic signal in the data of some patients. Highly accelerated deep learning-based reconstructions need to be carefully evaluated in the clinic, since artifacts may appear differently than with conventional reconstruction methods. Metrics often used in image reconstruction in addition to SNR, such as the structural similarity index (SSIM), could not be computed in the clinical data since we did not have a fully sampled scan. In a certain case reported in the database as a nonspecific white matter lesion, the CR deviated for high acceleration factors. We attribute this to measurement noise, since this case had minimally visible contrast and a bounding box including CSF with no signal. Our analysis was largely robust to such an effect by computing median values in bounding boxes. Future work could refine the distributed bounding boxes to segmented lesion maps to improve the accuracy in estimated quality metrics. Another limitation is that we could not compare with on-scanner reconstruction times since hardware and software differences hinder a comparison of the algorithm with the reconstructions performed on-scanner. Furthermore, a fully sampled reference could not be acquired, because of the risk of motion artifacts and image blurring in the 24–30 min scanning time of a sequence without acceleration. For the prospectively acquired patient data, no ground truth data were available, and comparisons with clinically used CS reconstructions prohibited an assessment of changes in diagnosis. However, the analysis of FastMRI data demonstrated that CR is preserved over a broad range of acceleration factors. The small but significant decrease of 4% in SSIM from four-fold to 12-fold acceleration only has a marginal impact on image quality.

We demonstrated the added value of deep learning in reconstructing 3D FLAIR scans in a clinically representative sample with neurological deficits. Reconstructions made with the physics-informed CIRIM model have increased SNRs and appear less noisy than PICS iterative reconstruction. The higher SNR in CIRIM reconstructions enables scanning at higher resolution. Moreover, the CIRIM achieves faster reconstruction times, which is crucial for the timely diagnosis of neurological deficits. Online inference on MRI scanners requires a graphical processing unit (GPU) to be installed on the reconstruction computer. The CIRIM, which balances the network size and is therefore memory efficient, does not place high demands on the specifications of GPU cards, requiring 1.6 GB of memory for 264 k parameters in total. This work shows the promise of physics-informed neural networks in accelerated MRI reconstruction. Future work should evaluate whether the surplus in the SNR can be traded for further acceleration.

## Supplementary Information

Below is the link to the electronic supplementary material.

Supplementary file1 (PDF 510 KB)
