# Supplementary material for: Deep learning for efficient reconstruction of highly accelerated 3D FLAIR MRI in neurological deficits
Source: MAGMA. 2024 Aug 30;38(1):1–12. doi: 10.1007/s10334-024-01200-8 (PMC11790796; doi:10.1007/s10334-024-01200-8)
Supplement: Supplementary file 1 — Supplementary file1 (PDF 510 KB) [file 10334_2024_1200_MOESM1_ESM.pdf]

Supplementary Information to the article "Deep learning for efficient reconstruction of highly accelerated 3D FLAIR MRI in neurological deficits" by Liebrand et al.

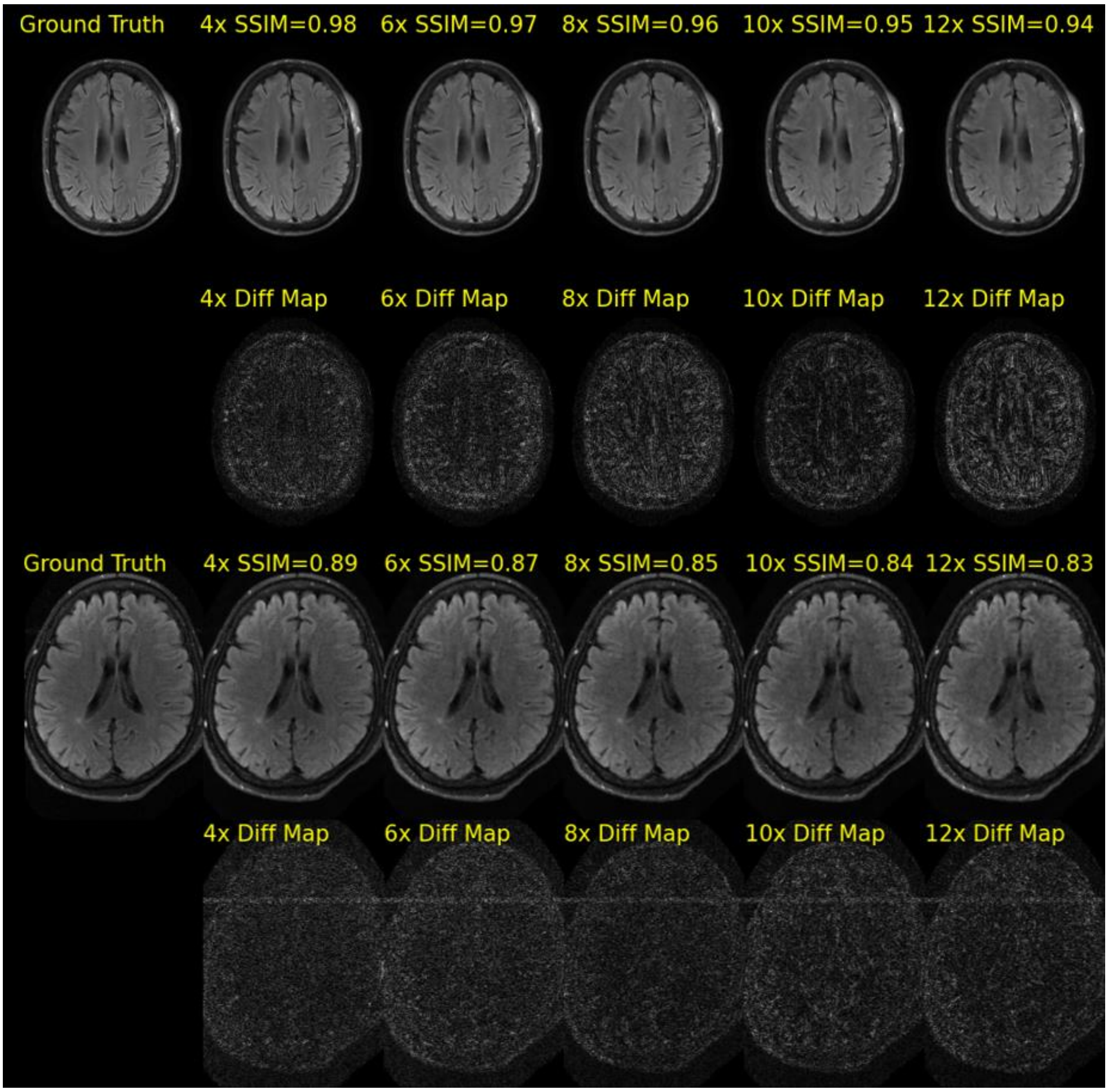

Supplementary Figure 1. Example reconstructions of FastMRI FLAIR scans with relatively high and low Structural Similary (SSIM) values, with absolute difference maps relative to the ground truth. Note that the line artefact in the lower scan is present in the ground truth and largely filtered out in the CIRIM reconstructions.
